# 3D printed elastomers with Sylgard-184-like mechanical properties and tuneable degradability†

**DOI:** 10.1039/d2py00113f

**Published:** 2022-03-30

**Authors:** Nevena Paunović, Jean-Christophe Leroux, Yinyin Bao

**Affiliations:** Institute of Pharmaceutical Sciences, Department of Chemistry and Applied Biosciences, ETH Zurich Vladimir-Prelog-Weg 3 8093 Zurich Switzerland jleroux@ethz.ch yinyin.bao@pharma.ethz.ch

## Abstract

The 3D printing of biodegradable elastomers with high mechanical strength is of great interest for personalized medicine, but rather challenging. In this study, we propose a dual-polymer resin formulation for digital light processing of biodegradable elastomers with tailorable mechanical properties comparable to those of Sylgard-184.

## Abstract 2

Digital light 3D printing of biodegradable elastomers with mechanical properties comparable to the ones of Sylgard-184 *via* dual-polymer resins.

## Graphical abstract

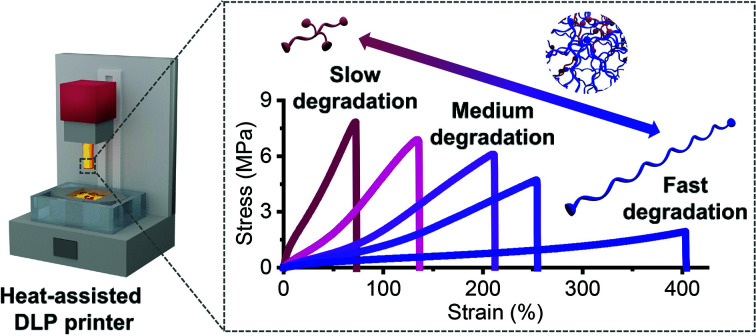

Additive manufacturing, commonly called 3D printing, is experiencing substantial development owing to its broad applications in academic research and industry.^1–6^ In particular, vat photopolymerization emerged as a powerful low-cost 3D printing technique, displaying high resolution and surface quality of the printed objects.^7–9^ Stereolithography (SLA)^10,11^ and digital light processing (DLP)^12,13^ are the two most commonly used vat photopolymerization techniques, both based on layer-by-layer crosslinking, with the latter possessing a micro-mirror device which can convert light spots into dynamic 2D patterns, resulting in faster printing. Jointly with computed tomography and other medical imaging tools, vat photopolymerization could be a game changer in personalized medicine by allowing for rapid production of highly-customized tissue scaffolds, implants and medical devices,^14–18^ especially when biodegradability is required.^19^ However, there are only a few examples of biodegradable photopolymers that are suitable for SLA or DLP 3D printing of elastomers with tuneable mechanical properties.^20–23^

In general, most of the biodegradable photopolymers designed for SLA or DLP (*e.g.*, acrylated poly(lactide) (PLA)^24,25^ or poly(ε-caprolactone) (PCL)^26^) have low molecular weights (MW, typically 600–3000 g mol^−1^) in order to keep the viscosity of the resin low, resulting in brittle 3D printed products (tensile strain <50%). Grijpma, Seppälä *et al.* showed that at higher MW, the photopolymers become solid and almost unprintable at room temperature.^24–26^ This challenge was overcome by Cohn *et al.* who realized the 3D printing of PCL methacrylate with MW of 10 000 g mol^−1^ in melt state by heat-assisted SLA.^27,28^ Due to semicrystallinity of PCL, the 3D printed products showed plasticity with a yield point at tensile strain of *ca.* 30%. Later on, Baker *et al.* diminished crystallization of PCL domains in order to produce liquid photopolymers by using random copolymerization of ε-caprolactone (CL) and trimethylene carbonate.^29,30^ Despite the improved elasticity of their DLP printed materials, these copolymers cannot provide high elasticity and mechanical strength at the same time. On the other hand, Becker *et al.* developed a series of poly(propylene fumarate) (PPF)-based photopolymers for 3D printing of biodegradable scaffolds.^31–33^ It was found that star-shape structure can remarkably reduce the viscosity of photopolymers, enabling the DLP printing of PPF with MW of 31 000 g mol^−1^.^32^ However, high concentrations of reactive diluents (30–50 wt%) were still needed for the resin preparation, limiting the mechanical tunability of these materials. Recently, we reported the 3D printing of a 4-arm copolymer, poly(d,l-lactide-*co*-ε-caprolactone) methacrylate (poly(DLLA-*co*-CL) MA) with MW of 15 000 g mol^−1^, using only 8.0 wt% of a reactive diluent.^22^ The printing was performed at 90 °C on a DLP printer with customized heat controller and resulted in an elastomeric material.^27,34^ It was found that the addition of a linear oligomer to the resin can highly enhance the mechanical properties of the 3D printed products due to the increased crosslinking density. Although their mechanical strength and stretchability are superior compared to previously reported 3D printed biodegradable elastomers, these resins still offer mechanical tunability in a relatively narrow range.

In this work, we investigate a new dual-polymer resin formulation using a linear poly(DLLA-*co*-CL) MA with high MW and a 4-arm oligomer to further develop advanced DLP printed biodegradable elastomers. By varying the feed ratio of two photopolymers, we can provide materials with highly-tuneable mechanical properties comparable with that of bulk Sylgard-184, a silicone elastomer commonly used for the manufacturing of biomedical devices (*e.g.*, microfluidic devices).^17,35^ Our 3D printed elastomers also showed formulation-dependent *in vitro* degradability, as well as excellent 3D printing quality.

The biodegradable poly(DLLA-*co*-CL)s were synthesized by ring-opening copolymerization of d,l-lactide (DLLA) and CL, in the melt with Sn(Oct)_2_ as a catalyst at 140 °C for 48 h (Fig. S1†).^22,36^ The resulted statistical copolymers possessed lower viscosity compared to homopolymers (*e.g.*, PCL or PLA) or block copolymers, which is pivotal for high-resolution DLP printing.^24,26,36^ In addition, random copolymerization can produce amorphous polymers that are needed for the fabrication of elastomeric objects. These polymers were further functionalized with methacrylate groups in order to make them photocrosslinkable (Fig. S1†).^22,36^ To maximize the mechanical tunability of the 3D printed products, we selected linear high MW photopolymer P1 (*M*_n,NMR_ = 14 700 g mol^−1^) and 4-arm oligomer P2 (*M*_n_ = 950 g mol^−1^) and formulated resins with different P1/P2 weight feed ratios, as shown in Scheme 1. P1 was synthesized using 2,2-diethyl-1,3-propanediol as an initiator with molar feed ratio DLLA/CL of 3/7, while P2 was synthesized with pentaerythritol and equimolar ratio of monomers. Both polymers were amorphous and liquid at room temperature, with glass transition temperatures of −38 and −40 °C, respectively (Fig. S2†).

**Scheme 1:**
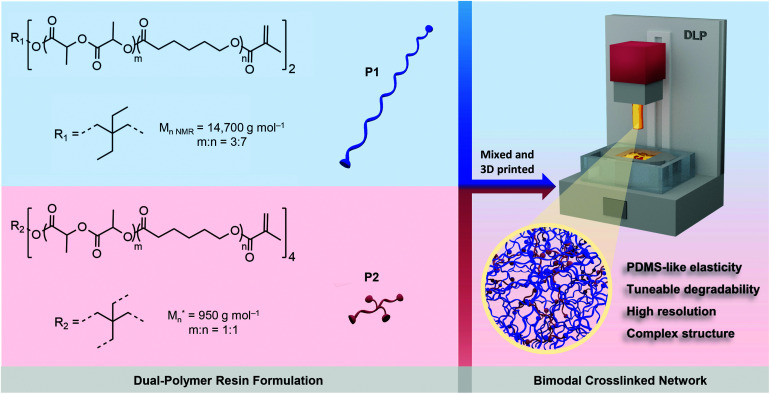
Schematic of the strategy for 3D printing of biodegradable elastomers using P1/P2 dual-polymer resins. Dual-polymer resins were designed by combining two distinct poly(DLLA-*co*-CL) MAs in various weight ratios. *M_n_ represents the highest peak of the MALDI-TOF spectrum of P2.

The combination of two distinct, but miscible, photopolymers should result in strong and elastic 3D printed materials with dominant properties depending on the P1/P2 weight ratio. P1 with its linear structure and long polymer chains can provide high flexibility, while P2 as the 4-arm oligomer should increase crosslinking density, and thus provide higher strength and modulus.^37–39^ Additionally, incorporating branched oligomers of very low viscosity (Fig. S2†) in high MW photopolymers can simultaneously reduce the viscosity of the resin, facilitating high-resolution DLP printing.

We formulated a series of P1/P2 dual-polymer resins with P2 weight fractions of 0, 10, 15, 20 and 30 wt%. All resins were prepared in the presence of a reactive diluent *N*-vinyl pyrrolidone (NVP, 8 wt%) in order to further reduce viscosity and dissolve the photoinitiator (phenylbis(2,4,6-trimethyl-benzoyl)phosphine oxide, 1 wt%) and the photoabsorber (Sudan I, 0.03 wt%). The formulation also contained a radical inhibitor (vitamin E, 0.3 wt%).

As expected, the addition of P2 to P1 remarkably reduced the viscosity of the resin (Fig. 1A). At the printing temperature (*ca.* 85 °C), viscosity decreased from 13.4 to 3.5 Pa s when the P2 fraction increased from 0 to 30 wt% (Fig. 1A).

**Fig. 1:**
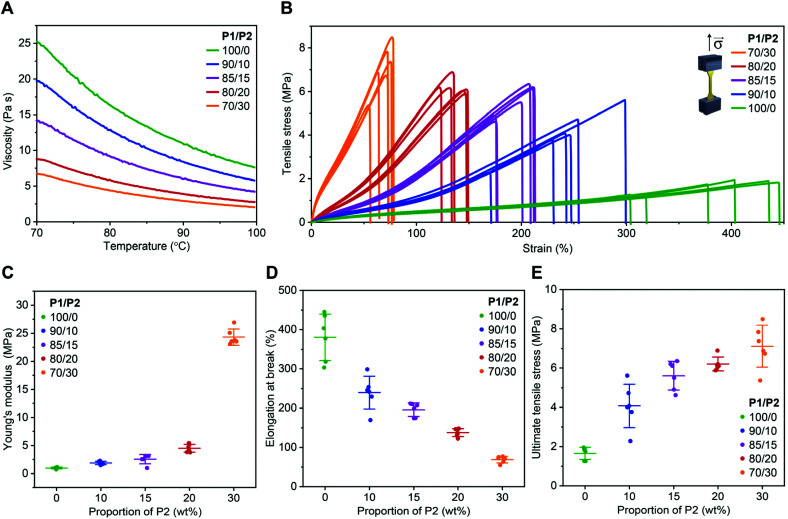
Viscosity of dual-polymer resins with various P1/P2 weight ratios and mechanical performance of the corresponding 3D printed materials. (A) Viscosity of the resins with various P1/P2 weight ratios. (B) Engineering tensile stress–strain curves of 3D printed elastomers using resins with various P1/P2 weight ratios. (C–E) Average Young's modulus (C), elongation at break (D), and ultimate tensile stress (E) of 3D printed elastomers using resins with various P1/P2 weight ratios extracted from tensile stress–strain curves. Mean ± s.d. (*n* = 6).

All resins enabled successful printing at 85 °C on a commercial DLP printer with a customized heat function. The 3D printed objects were amorphous with glass transition temperature between −38 and −36 °C (Fig. S3†). Similar thermal behaviour might be explained by the P1 dominance in the investigated dual-polymer systems, as well as by the fact that P1 and P2 are both amorphous polymers with similar glass transition temperatures. The mechanical performance of these materials was evaluated in a tensile test using dog bone-shaped samples. The 3D printed specimens from P1-only resin showed excellent elasticity (Fig. 1B), with Young's modulus of *ca.* 1.0 MPa (Fig. 1C) and elongation at break of *ca.* 380% (Fig. 1D), while ultimate tensile stress was 1.7 MPa (Fig. 1E). A gradual increase of P2 fraction up to 20 wt%, resulted in an increased Young's modulus and strength, while preserving elasticity. For example, at 15 wt%, the Young's modulus was of 2.5 MPa with elongation at break of 200%. The material also became much stronger than the P1-only one, with ultimate tensile stress of 6.2 MPa, as shown in Fig. 1B–E.

A further increase of the P2 fraction in the photopolymer resin to 30 wt% resulted in a stiffer product with Young's modulus of *ca.* 24 MPa and elongation at break of *ca.* 70%, while ultimate tensile stress was *ca.* 7 MPa (Fig. 1B–E). A similar phenomenon has been observed with the crosslinking networks based on poly(trimethylene carbonate)^41^ or poly(dimethylsiloxane) (PDMS).^42^ The observed trend was in accordance with higher crosslinking density brought by the higher P2 content, as indicated by the higher gel fraction^40^ from the dual-mode networks (Fig. S4†). The swelling of the 3D printed products in THF further confirmed the P2 fraction-dependent crosslinking density (Fig. S4†).

Note that these 3D printed biodegradable elastomers are superior compared to the previously reported materials,^26–32^ regarding their elasticity, strength and stretchability. Interestingly, we found that the dual-polymer system showed mechanical properties comparable with that of bulk Sylgard-184 — a typical PDMS elastomer, at different curing temperatures (25–200 °C),^35^ while having the additional feature of being biodegradable. This may grant them promise as an alternative material to medical PDMS when bioresorbability of a medical device is desired.

To evaluate the impact of P2 weight fraction on the degradation profiles of the 3D printed products, we selected three formulations (P1/P2 of 100/0, 85/15 and 70/30, w/w) and printed tubular objects (H 10.0 mm, ∅ 7.2 mm, thickness 1.0 mm) for the degradation test. We incubated these specimens separately in phosphate buffered saline (PBS) pH 7.4 at 50 °C and followed their weight and morphology over time. As shown in Fig. 2A, at the beginning of the study, all three compositions showed neglectable weight loss (*ca.* 4% until week 3). Later on, the degradation rate rapidly increased in a P1/P2 feed ratio-dependent manner, with P1-only objects degrading the fastest and the ones with 30 wt% P2 the slowest. This trend in weight loss was also observed in water uptake. The P1-only object absorbed water much faster than the ones based on P1/P2 85/15 and 70/30, w/w (Fig. 2B). This is in accordance with gel fraction and swelling ratio of the 3D printed products in THF (Fig. S4†). This behaviour is typical for polyester networks, as the diffusion of the hydrolysed products from objects of high thickness is slower than the hydrolysis of ester bonds. With the accumulation of acidic products overtime, a local acidic environment is generated catalysing the degradation.^36,43,44^ This was manifested as a slight, and then more pronounced decline in pH of the buffer over time (Fig. S5†).

**Fig. 2:**
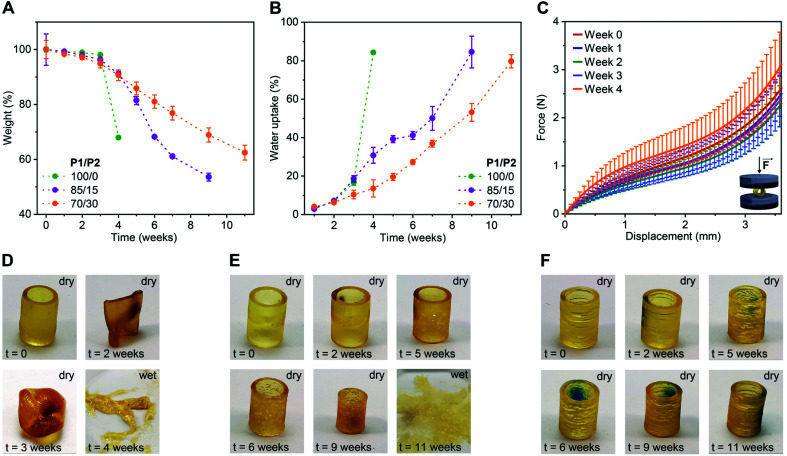
Degradation performance of 3D printed tubular objects based on P1/P2 dual-polymer resins (100/0, 85/15 and 70/30, w/w) in PBS pH 7.4 at 50 °C. (A and B) Changes in dry weight (A) and water uptake (B) of 3D printed tubes (H 10.0 mm, ∅ 7.2 mm, thickness 1.0 mm) over time. Mean ± s.d. (*n* = 4). (C) Average force-displacement curves obtained in compression tests with dry tubular objects based on P1/P2 85/15, w/w polymer blend at different time points. Mean ± s.d. (*n* = 3). (D–F) Photographs of a representative tubular object based on P1/P2 100/0, w/w (D), P1/P2 85/15, w/w (E), and P1/P2 70/30, w/w (F) during the degradation study.

To study the effect of degradation on the mechanical performance of the 3D printed products, we conducted uniaxial compressive test using the tubular objects based on P1/P2 85/15, w/w. In accordance with the weight loss, the hydrolysis of ester bonds was slow during first three weeks of incubation and did not affect mechanical strength (Fig. 2C and S6†). At week 4, the tested objects showed increased resistance, which is likely due to the degradation-related slight decrease in object dimensions.

The change in dimensions and surface of the objects over time were visually monitored as shown in Fig. 2D–F and S7.† The P1-only object became very sticky and even more elastic after two weeks, and progressed to a full collapse of the structure after four weeks of degradation (Fig. 2D and S7†). This is consistent with our previous report.^34^ The P1/P2 dual-polymer resins resulted in more stable products under the tested conditions and preserved their shape over the period of two months. The dimensions of P1/P2 85/15, w/w-based objects started to decrease from week 4 on, while P1/P2 70/30, w/w-based ones preserved their dimensions for seven weeks (Fig. 2E, F and S7†). These results indicated highly tuneable degradability of the 3D printed elastomers based on the dual-polymer resin strategy. The objects based on P1-only and P1/P2 85/15, w/w dual-polymer resins finally degraded to soft hydrogels after 4 and 11 weeks, respectively (Fig. 2D and E), while P1/P2 70/30, w/w-based objects maintained tubular shape during the tested period (Fig. 2F).

To determine whether residual double bonds persisted in the 3D printed networks, we compared the FTIR spectra of the resins with that of their corresponding 3D printed products (Fig. S8†). Complete crosslinking of vinyl groups was observed in all 3D printed objects (Fig. S8†), minimizing the risk of methacrylate-related cytotoxicity.^45^ In our previous study, we already showed in rabbits that DLP printed airway stents prepared with poly(DLLA-*co*-CL) methacrylates were biocompatible,^22^ suggesting that other DLP printed materials based on this copolymer might be safe as well. However, in future work, it will be important to characterize the products generated upon the degradation of the dual-poly(DLLA-*co*-CL) methacrylate systems.

To further demonstrate the suitability of our dual-polymer resins for various applications where high precision is prerequisite, we printed different objects with complex architectures using P1/P2 85/15, w/w resin (Fig. 3). Three distinct objects containing gyroid, hexagonal prism, and cantitruncated cubic structure were successfully printed (Fig. 3A). All of them showed smooth surface, excellent shape fidelity and good elasticity (Fig. 3B and C). The minimal feature size was determined to be about 80 μm, indicating the high resolution printing with DLP.

**Fig. 3:**
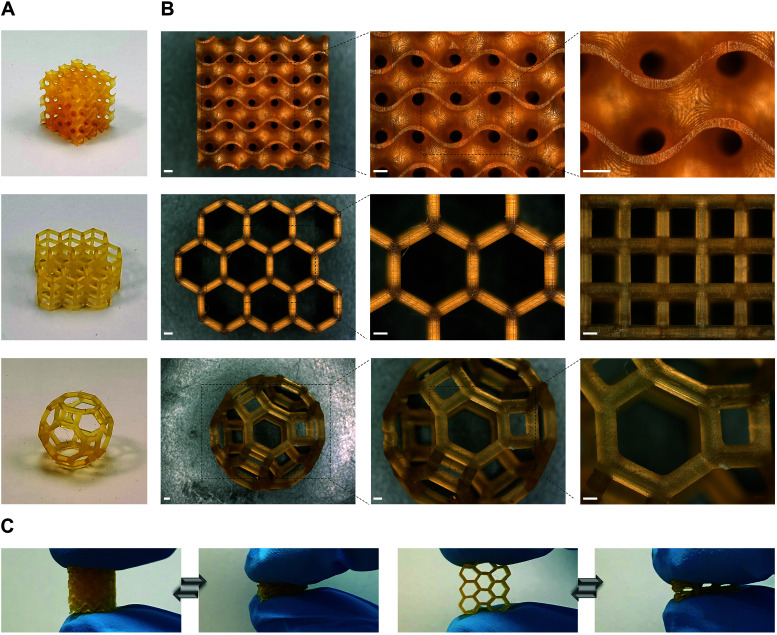
High resolution 3D printing with P1/P2 85/15, w/w dual-polymer resin. (A and B) Pictures (A) and corresponding microscopy images (B) of three 3D printed objects with complex architectures. Scale bars 500 μm. (C) The 3D printed objects showing high elasticity indicated by finger compression.

## Conclusions

In summary, we report the high resolution DLP printing of biodegradable elastomers with tuneable mechanical properties and degradability *via* dual-polymer resin formulation. By adjusting the weight feed ratio of high to low MW photopolymers in the resin, we could 3D print objects with elasticity (Young's modulus 1.0–24 MPa) comparable to Sylgard-184. They also showed superior mechanical strength (1.7–7.0 MPa) and elongation at break (70–380%) compared to reported biodegradable networks produced by SLA/DLP. The 3D printed objects exhibited tuneable *in vitro* degradation kinetics in an accelerated degradation study, with two compositions eventually degrading into soft hydrogels. The proposed dual-polymer strategy in design of biomedical resins could offer tuneable elasticity, bioresorbability and excellent mechanical properties of 3D printed objects, thus potentially expanding the applications of photopolymerization 3D printing in the rapid fabrication of personalized implants and medical devices.

## Conflicts of interest

There are no conflicts of interest to declare.

## Supplementary Material

PY-013-D2PY00113F-s001

PY-013-D2PY00113F-s002
