# Supplementary material for: 3D printed elastomers with Sylgard-184-like mechanical properties and tuneable degradability
Source: Polym Chem. 2022 Mar 30;13(16):2271–6. doi: 10.1039/d2py00113f (PMC9016719; doi:10.1039/d2py00113f)
Supplement: PY-013-D2PY00113F-s002 [file PY-013-D2PY00113F-s002.pdf]

Electronic Supplementary Material (ESI) for Polymer Chemistry.
This journal is © The Royal Society of Chemistry 2022

Supporting information

for

3D Printed Elastomers with Sylgard-184-like Mechanical Properties and Tuneable Degradability

Nevena Paunović, Jean-Christophe Leroux,* Yinyin Bao*

Institute of Pharmaceutical Sciences, Department of Chemistry and Applied Biosciences, ETH Zurich, Vladimir-Prelog-Weg 3, 8093 Zurich, Switzerland.

E-mail: jleroux@ethz.ch; yinyin.bao@pharma.ethz.ch

Experimental

Materials

Pentaerythritol, triethylamine, magnesium sulphate ($MgSO_4$), tetrahydrofuran (THF; extra dry), toluene (extra dry) and acetone (extra dry) were purchased from Acros Organics. ε-caprolactone (CL) was obtained from Tokyo Chemical Industry. 3,6-Dimethyl-1,4-dioxane-2,5-dione (DLLA) was purchased from Huizhou Foryou Medical Devices Co. Ltd. or Acros Organics. Methacryloyl chloride was bought from Acros Organics or Sigma-Aldrich. Tin(II)-2-ethylhexanoate [$Sn(Oct)_2$], 2,2-diethyl-1,3-propanediol, sodium bicarbonate ($NaHCO_3$), sodium chloride (NaCl), (+)-α-tocopherol (vitamin E), N-vinylpyrrolidone (NVP), phenylbis(2,4,6-trimethyl-benzoyl)phosphine oxide (BAPO), 1-(phenyldiazenyl)naphthalen-2-ol (Sudan I), phosphate buffered saline (PBS) 7.4 (tablets), methanol, 2-propanol and lithium bromide (LiBr) were purchased from Sigma-Aldrich. Hexane, dichloromethane (DCM) and dimethylformamide (DMF) were purchased from Thermo Fisher Scientific. Dimethyl sulfoxide-d6 (DMSO-d6) was obtained from Sigma-Aldrich or Apollo Scientific. All chemicals were used as received.

Polymer synthesis

Poly(D,L-lactide-*co*-ε-caprolactone)s (poly(DLLA-*co*-CL)s) were synthesized by ring-opening polymerization of DLLA and CL in melt, with 2,2-diethyl-1,3-propanediol (P1) or pentaerythritol (P2) as an initiator and $Sn(Oct)_2$ as a catalyst (**Fig. S1**).[1] For P1, DLLA/CL molar feed ratio was 3/7, while for P2 equimolar ratio of monomers was used. Afterwards,

the synthesized poly(DLLA-*co*-CL)s were functionalized with methacryloyl chloride in THF with triethylamine as proton scavenger (**Fig. S1**).[1]

Synthesis of P1: 2,2-diethyl-1,3-propanediol (0.344 g, 2.6 mmol), CL (25.22 g, 221 mmol) and DLLA (13.55 g, 94 mmol) were added to a Schlenk flask and placed under vacuum for *ca.* 1.5 h in order to remove water and oxygen. Then, the flask was purged with argon to create an inert atmosphere and inserted in an oil bath at 140 °C to let lactide melt. Thereafter, the flask was removed from the oil bath, three vacuum-argon cycles were performed and $Sn(Oct)_2$ (8 µL, 0.026 mmol) in extra dry toluene (22 µL) was added under the argon. The reaction mixture was then stirred in an oil bath at 140 °C for 48 h. The resulted viscous copolymer was dissolved in THF and precipitated in hexane. Based on ^1H NMR spectroscopy, the conversion of DLLA and CL were 89-96 and 98-99%, respectively (**Fig. S9**) and polydispersity index based on size exclusion chromatography (SEC) was 1.37 (**Table S1**). This copolymer was further dissolved in extra dry THF (250 mL) and after the addition of triethylamine (2.9 mL, 20.8 mmol), the mixture was purged with argon for 15 min. Thereafter, methacryloyl chloride (2.0 mL, 20.8 mmol) in extra dry THF (*ca.* 4 mL) was added dropwise, while stirring, and the reaction was left at RT for 24 h. Afterwards, the salts were removed by centrifugation (4000 *g*, 4 °C, 30 min) and vitamin E (0.20 g) was added to the supernatant to prevent premature crosslinking. The polymer solution was then concentrated under vacuum and later precipitated in methanol. The resulted viscous polymer was dried under high vacuum for 5-7 days. Based on ^1H NMR spectroscopy, the conversion of hydroxyl end groups was *ca.* 54% (**Fig. S9**).

Synthesis of P2: pentaerythritol (6.26 g, 46 mmol), CL (10.50 g, 92 mmol) and DLLA (13.26 g, 92 mmol) were added to a Schlenk flask and placed under vacuum for *ca.* 1.5 h. The synthesis then followed the same protocol as for unfunctionalized P1, with the only exception of adding higher amount of the catalyst - $Sn(Oct)_2$ (150 µL, 0.46 mmol) without toluene. Based on ^1H NMR spectroscopy, the conversion of DLLA and CL were 40 and *ca.* 100%, respectively (**Fig. S10**). This copolymer was then dissolved in extra dry THF (100 mL) and triethylamine (28.9 mL, 207 mmol) was added. The solution was further purged with argon for 15 min. Afterwards, methacryloyl chloride (20 mL, 207 mmol) in extra dry THF (*ca.* 20 mL) was added dropwise and the reaction was performed for 24 h at RT. The suspension was centrifuged (4000 *g*, 4 °C, 30 min) and vitamin E (0.12 g) was added to the supernatant. The polymer solution was then concentrated under vacuum and precipitated in hexane. After drying, the polymer was dissolved in DCM (200 mL), washed with saturated

$NaHCO_3$ and NaCl aqueous solutions, and then dried with anhydrous $MgSO_4$. The solution was again concentrated after the addition of vitamin E (0.10 g), precipitated in hexane for the second time, and then dried under high vacuum for 5-7 days. ^{1}H NMR spectroscopy (**Fig. S10**) could not be used for calculating the conversion of hydroxyl end groups, as after the precipitation in hexane, molecular weight distribution shifted to higher values, which was confirmed by MALDI-TOF (**Fig. S11**).

Polymer characterization

^{1}H NMR spectra were recorded in DMSO-d6 on Bruker AV400 spectrometer at 400 Hz. SEC was performed on a Viscotek TDAmax system with two Viscotek columns [D3000, poly(styrene-*co*-divinylbenzene)] and differential refractive index detector (TDA 302, Viscotek). The sample was dissolved in DMF, filtered using the 0.2-µm syringe filter (polytetrafluoroethylene, PTFE) and eluted with DMF with 0.1 wt% of LiBr (mobile phase flow, 0.5 ml min^{-1}). The macromolecular characteristics were determined relative to a poly(methyl methacrylate) standard curve (PSS Polymer Mainz; 2500 to 89, 300 g mol^{-1}). Differential scanning calorimetry (DSC) analysis was carried on TA Q200 DSC (TA Instruments–Waters LLC). The samples (*ca.* 10 mg) were tested in Tzero hermetic pans (TA Instruments–Waters LLC) in heat-cool-heat cycles from -80 to 200 °C under nitrogen flow (50 mL min^{-1}) with heating and cooling rates of 10 °C min^{-1}. Data were analyzed using TA Instruments Universal Analysis 2000 software (5.5.3). Viscosity measurements were performed using a HAAKE RheoStress 600 rotational rheometer (Thermo Electron Corporation) with cone and plate geometry (35 mm/2°). Viscosity was recorded at a shear rate of 100 s^{-1} from 70 to 100 °C, with a heating rate of 0.05 °C s^{-1} and Thermogap function enabled. Data were analyzed by RheoWin Data Manager (Thermo Electron Corporation). MALDI-TOF spectra were measured on a Bruker Daltonics Ultraflex II spectrometer in the positive ion reflector mode using trans-2-[3-(4-tert-butylphenyl)-2-methyl-2-propenylidene] as a matrix.

DLP 3D printing

Resins were prepared by dissolving the photoinitiator (BAPO, 1.0 wt%) and the photoabsorber (Sudan I, 0.03 wt%) in reactive diluent (NVP, 8.0 wt%) and adding the solution to the mixture of photopolymers with antioxidant (vitamin E, 0.3 wt%). The resins were then sonicated at 60 °C until they became homogenous. A commercial DLP 3D printer (Asiga PICO2) with the light source of 405 nm equipped with a customized resin tray and

printing head with heating functions was used for all printings. The printing temperature was 85 °C, while exposure time and initial exposure time were 3.3 and 15 s, respectively. After printing, the printed objects were washed in acetone and 2-propanol and then cured in an Asiga Pico Flash UV chamber for 30 min.

Gel fraction and swelling ratio

Three cuboids (5 x 5 x 1 mm) were 3D printed with each of three tested resins (P1/P2 100/0, 85/15 and 70/30, w/w). They were placed separately in closed glass vials with 5 mL THF and incubated at room temperature. After 24 h, the samples were taken out and quickly blotted with a paper tissue and then left for drying under vacuum at RT to constant mass. The gel fraction (wt%) and swelling ratio (wt%) were calculated using Eqs. 1 and 2, respectively[2]

$$gel\,fraction = \frac{wt_{dry}}{wt_0} x100 \quad (1)$$

$$swelling\,ratio = \frac{(wt_{wet} - wt_{dry})}{wt_{dry}} x100 \quad (2)$$

where wt_0 is the initial weight of a 3D printed cuboid after the printing, cleaning and curing, wt_{wet} is the mass of a cuboid in a wet state after the quick blotting and wt_{dry} is the mass of a completely dried object.

Mechanical characterization of 3D printed objects

Tensile tests were performed on 3D printed dog bone-shaped specimens using an AGS-X (Shimadzu) universal testing machine with 100-N load cell. The gauge length was set to 13 mm and a testing rate was 20 mm min^{-1}. Engineering stress (σ) and strain (ε) were calculated using Eqs. 3 and 4, respectively[3]

$$\sigma = \frac{F}{A_0} \quad (3)$$

$$\varepsilon = \frac{L}{L_0} x100 \quad (4)$$

where F is the force, A_0 is the initial cross-sectional area, L is the elongation, and L_0 is the initial gauge length. Young's modulus was calculated as a slope of the initial linear region (first 10 points) of tensile stress-strain curves.

Degradation study

Four tubular objects (H 10.0 mm, Ø 7.2 mm, thickness 1.0 mm) were 3D printed with each of three tested resins (P1/P2 100/0, 85/15 and 70/30, w/w). They were placed separately in closed Falcon tubes with 50 mL PBS buffer pH 7.4 and incubated at 50 °C. At each time point, the specimens were taken out, rinsed with deionized water, wiped with paper tissue and dried under vacuum at 50 °C to constant weight, while the medium was replaced with a fresh one. The water uptake (wt%) was calculated using Eq. 5

$$\text{water uptake} = \frac{wt_{wet} - wt_{dry}}{wt_{dry}} \times 100$$

(5)

where wt_{wet} is the mass of a tubular object in a wet state after the wiping with a paper tissue and wt_{dry} is the mass of a stent in a dry state after drying under the vacuum. pH was measured with InLab pH electrode (Mettler Toledo) on Orion Dual Star pH/ISE Meter (Thermo Fisher Scientific) at room temperature. Three tubular objects based on P1/P2 100/0, w/w collapsed at week 4 and that time point is represented with only one data point. One specimen of P1/P2 85/15, w/w collapsed under external force when transferring the object on week 9, and that time point includes three specimens.

Uniaxial compression tests were performed with three tubular objects based on P1/P2 85/15, w/w in dry state using TA.XTplus texture analyzer (Stable Micro Systems) with 100-N load cell at the compression rate of 12 mm min^{-1}. Every sample was tested three times.

Microscopy

3D printed objects of complex architectures were visualized using a Keyence scanning laser microscope (Keyence Corporation).

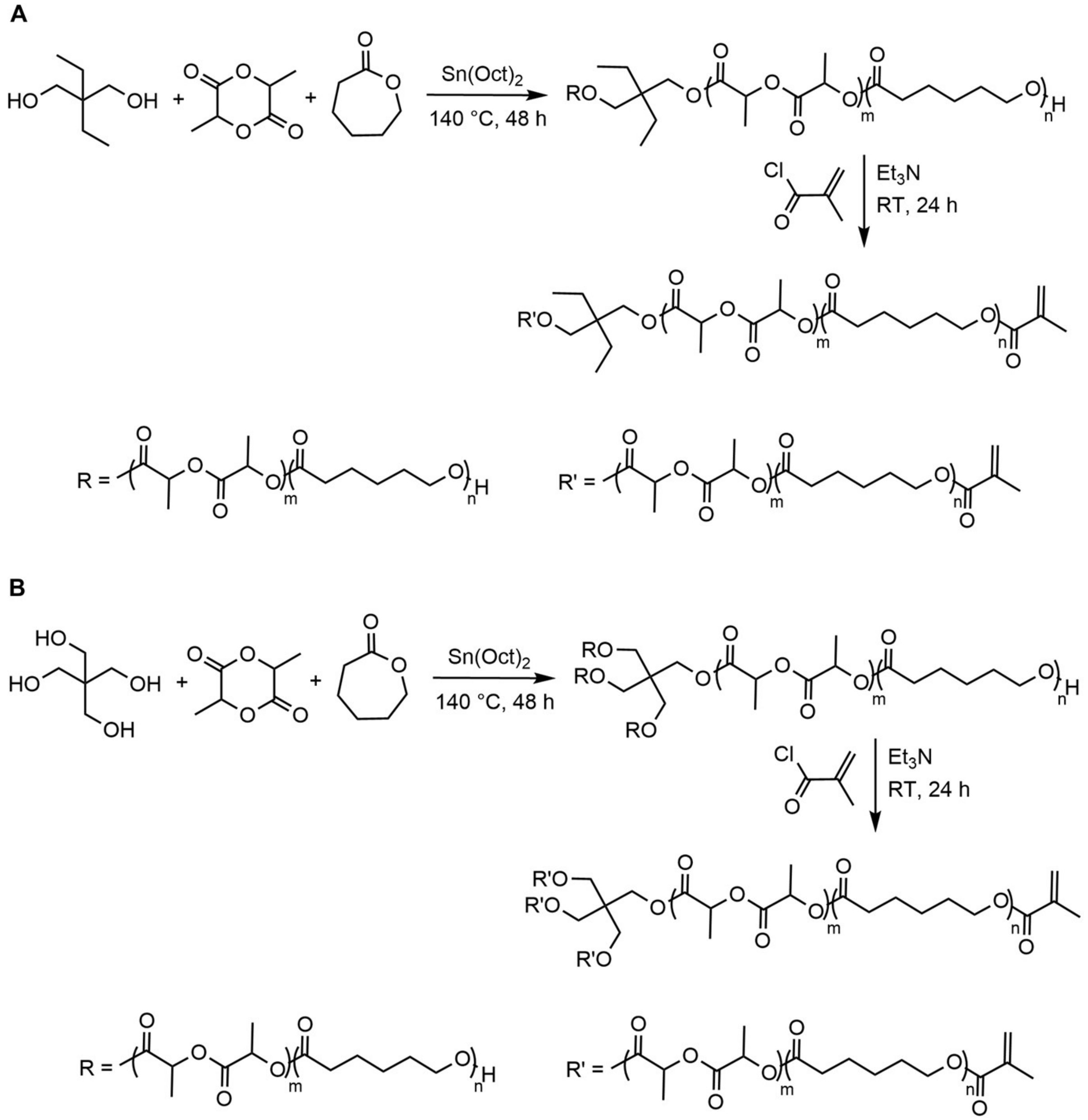

Figure S1. Syntheses of poly(DLLA-*co*-CL) methacrylates. (**A**) Linear (m/n = 3/7, $M_{n\ NMR}$ = 14,700 g mol^{-1}). (**B**) 4-arm (m/n = 5/5, M_n^* = 950 g mol^{-1}). $^*M_n$ represents the highest peak of the MALDI-TOF spectrum.

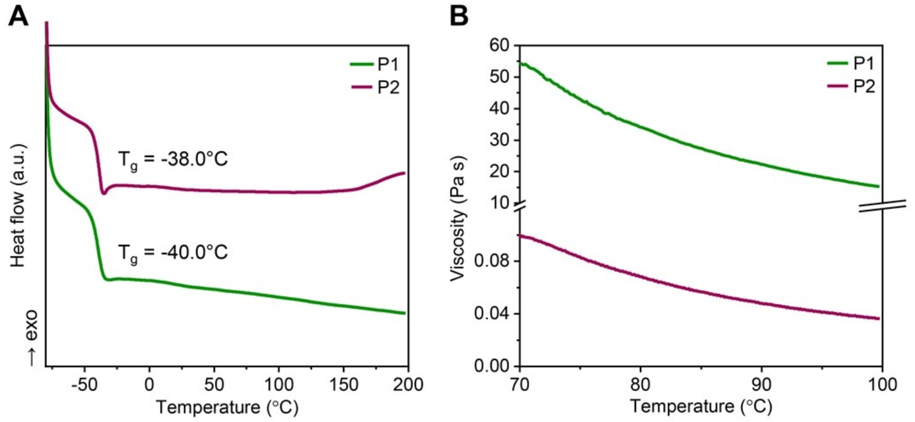

Figure S2. Characterization of P1 and P2 photopolymers. (**A**) DSC curves from the second heating cycle obtained in a heat-cool-heat mode. Glass transition temperature (T_g) from this cycle is presented on the graph. (**B**) Viscosity.

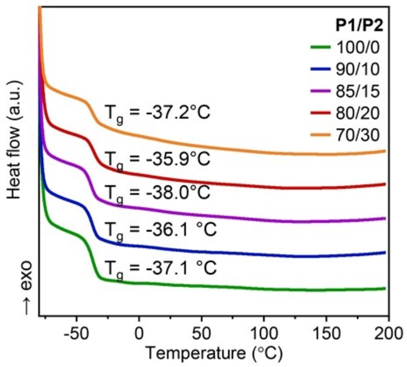

Figure S3. DSC heating curves of DLP printed materials with various P1/P2 weight ratios. DSC curves from the second heating cycle obtained in a heat-cool-heat mode. Glass transition temperature (T_g) from this cycle is presented on the graph.

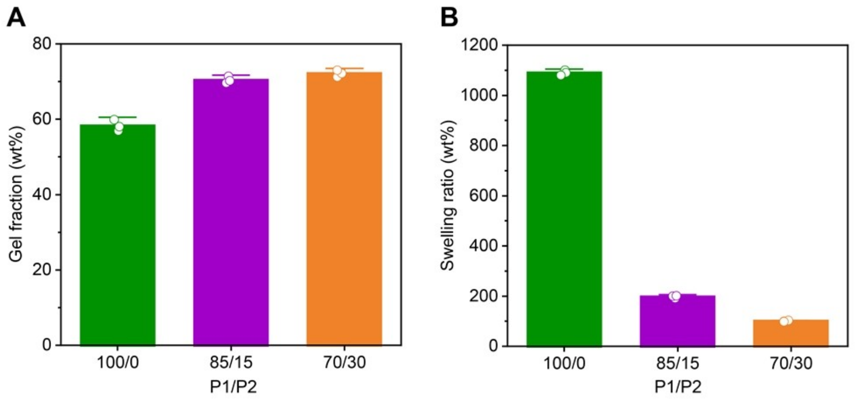

Figure S4. Impact of P2 on gel fraction and swelling ratio of 3D printed materials. (**A**) Gel fraction and (**B**) swelling ratio of dual-polymer P1/P2-based materials (100/0, 85/15 and 70/30, w/w). Test was performed in THF with 3D printed cuboids (5 x 5 x 1 mm) for 24 h. Mean + s.d. (n = 3).

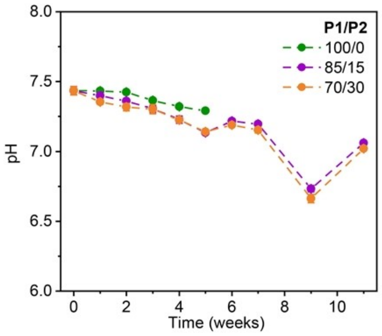

Figure S5. Change in pH of the incubation medium during the degradation study in PBS pH 7.4 at 50 °C. Materials tested were based on P1/P2 100/0, 85/15 and 70/30, w/w. Mean ± s.d. (n = 4).

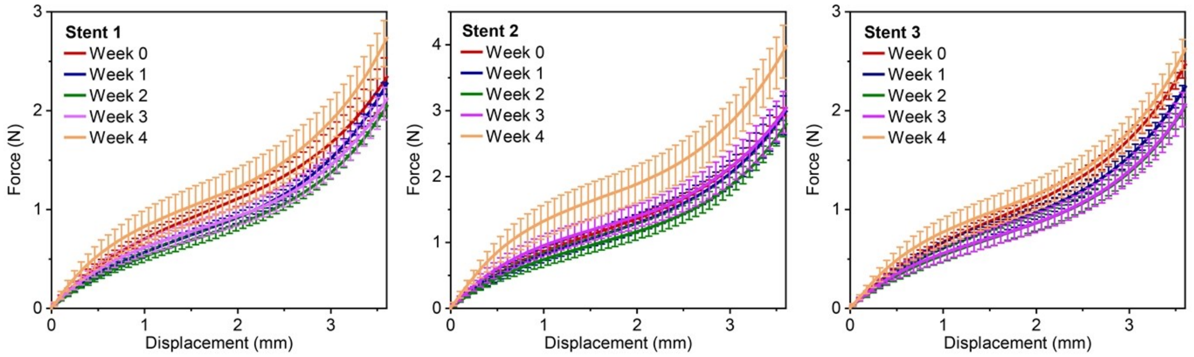

Figure S6. Change in mechanical performance of tubular objects during the degradation study. Average force-displacement curves obtained in compression tests with dry tubular objects based on P1/P2 85/15, w/w polymer blend at different time points. Every stent was compressed three times at every time point. Mean ± s.d. (n = 3).

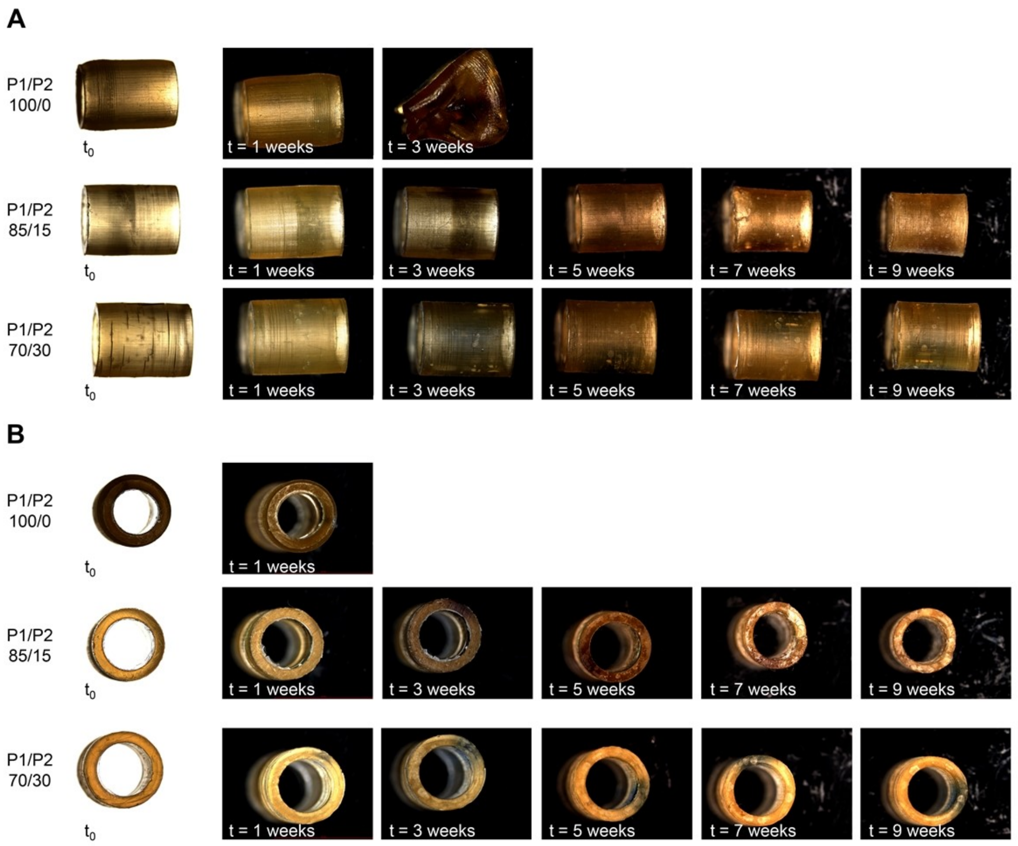

Figure S7. Changes in dimensions and surface morphology during the degradation study. Photographs of representative tubular objects based on P1/P2 100/0, 85/15 and 70/30, w/w at different time points. (**A**) Side view. (**B**) Top view.

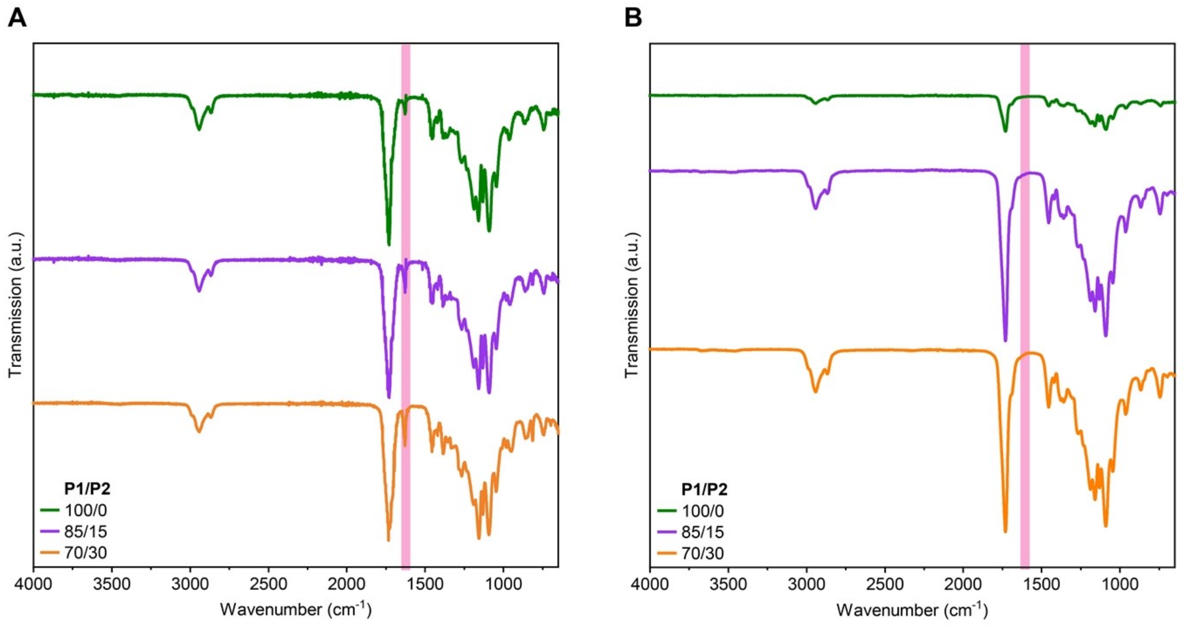

Figure S8. **FTIR spectra of P1/P2 dual-polymer systems.** FTIR spectra of the resins (A) and their corresponding 3D printed objects (B). Pink band marks the position of the double bonds. Double bonds are not observed in panel B.

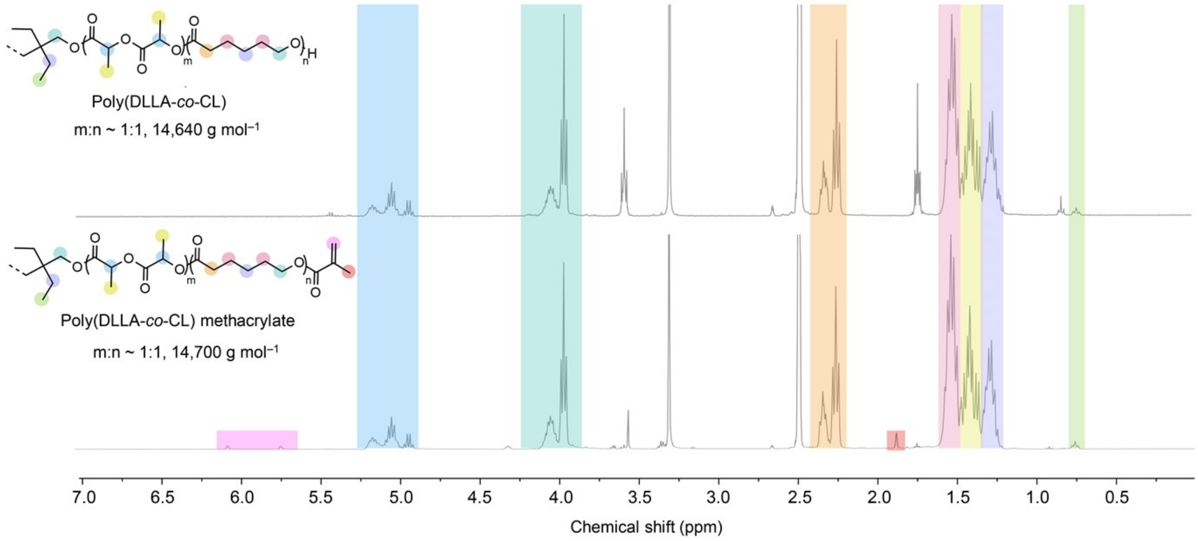

Figure S9. ^{1}H NMR spectra of P1 with linear structure before and after functionalization. ^{1}H NMR spectra of unfunctionalized P1 (upper) and P1 (lower) in DMSO-d6. Solvent peaks appeared at 2.50 ppm (DMSO), 3.33 ppm (H_2O), 3.60 and 1.76 ppm (THF). The signals of unreacted DLLA and CL located at 5.3-5.5 and 1.25, and 2.7 ppm, respectively.

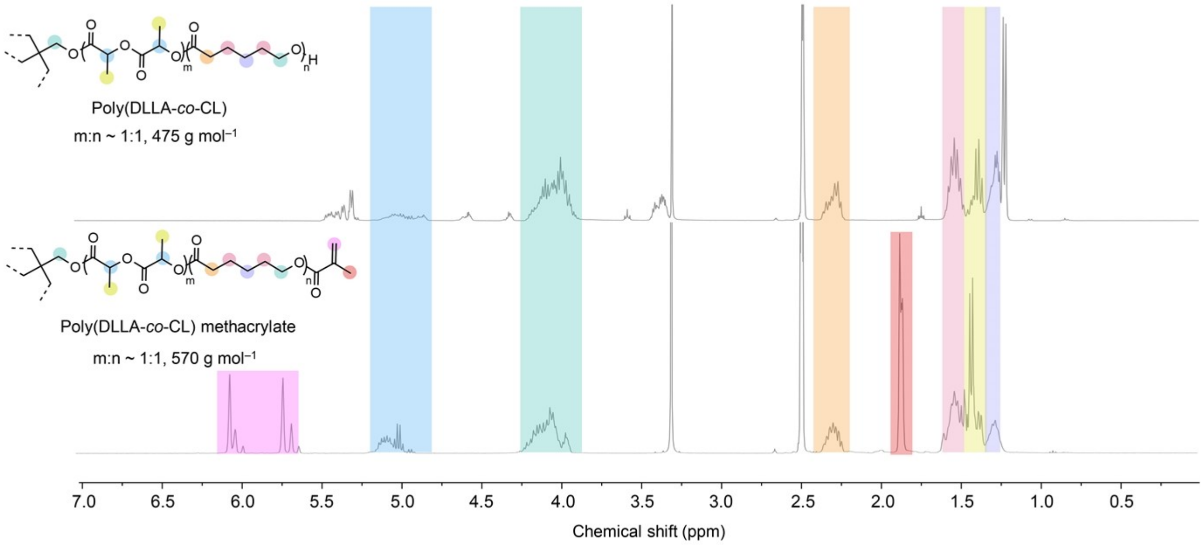

Figure S10. ^{1}H NMR spectra of P2 with 4-arm structure before and after functionalization. ^{1}H NMR spectra of unfunctionalized P2 (upper) and P2 (lower) in DMSO-d6. Solvent peaks appeared at 2.50 ppm (DMSO), 3.33 ppm (H_2O), 3.60 and 1.76 ppm (THF). The signals of unreacted DLLA and CL located at 5.3-5.5 and 1.25, and 2.7 ppm, respectively.

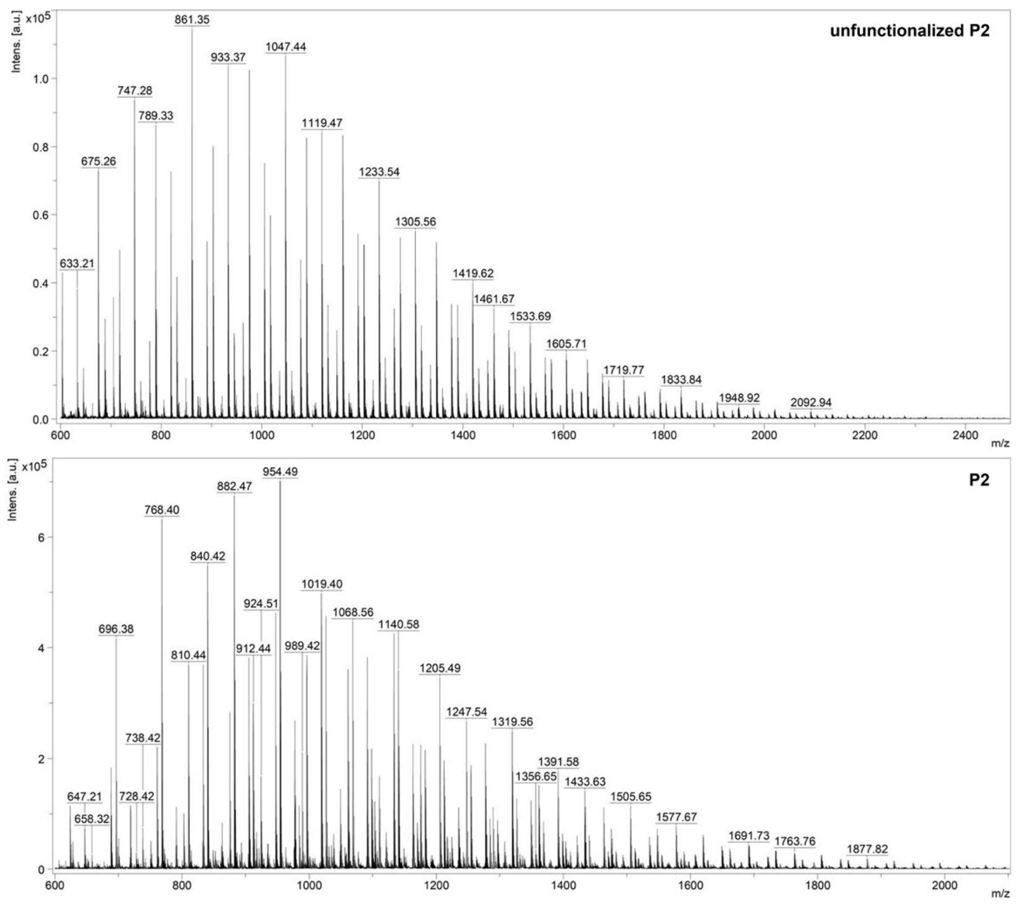

Figure S11. MALDI-TOF mass spectra of P2. MALDI-TOF mass spectra of P2 and its unfunctionalized polymer.

Table S1. Characterization of P1 photopolymer before functionalization.[a)]

Polymer	$M_{n\ NMR}$[b)] (g mol^{-1})	[CL]/[DLLA][c)] (mol/mol)	$M_{n\ SEC}$[d)] (g mol^{-1})	*Đ*[d)]
P1	14,600	83/35	30,655	1.37

[a)] Unfunctionalized polymer was used for SEC experiments to prevent potential crosslinking on the columns.

[b)] Molecular weight of unfunctionalized polymer calculated from NMR spectra based on the conversion of DLLA ($DLLA_{\%}$) and CL ($CL_{\%}$) by using Eq. 1, 2, and 3

$$DLLA_{\%} = \frac{A_{5.2\ \text{ppm}}}{A_{5.2\ \text{ppm}} + A_{5.4\ \text{ppm}}} \times 100 \quad (1)$$

$$CL_{\%} = \frac{A_{2.3\ \text{ppm}}}{A_{2.3\ \text{ppm}} + A_{2.7\ \text{ppm}}} \times 100 \quad (2)$$

$$M_n = MW_{initiator} + \frac{N_{DLLA} \times DLLA_{\%}}{100} \times MW_{DLLA} + \frac{N_{CL} \times CL_{\%}}{100} \times MW_{CL} \quad (3)$$

where A is the peak integral at a specific chemical shift, and N is the number of equivalents of a monomer used in the synthesis.[4]

[c)] Degree of polymerization calculated from NMR spectra based on comparison of equivalents of the monomers in the polymer obtained by multiplying conversion of the monomers ($DLLA_{\%}$ and $CL_{\%}$) with corresponding initial number of equivalents (N_{DLLA} and N_{CL}).[4]

[d)] Number averaged molecular weight (M_n) and polydispersity index (Đ) calculated from SEC spectra.

References

1 B. G. Amsden, G. Misra, F. Gu and H. M. Younes, *Biomacromolecules*, 2004, **5**, 2479–2486.

2 S. Wang, D. H. Kempen, N. K. Simha, J. L. Lewis, A. J. Windebank, M. J. Yaszemski and L. Lu, *Biomacromolecules*, 2008, **9**, 1229–1241.

3 W. F. Hosford, *Solid Mechanics*, Cambridge University Press, 2010.

4 N. Paunović, Y. Bao, F. B. Coulter, K. Masania, A. Karoline, K. Klein, A. Rafsanjani, J. Cadalbert, P. W. Kronen, A. Karol, Z. Luo, F. Rüber, D. Brambilla, B. Von, D. Franzen, A. R. Studart and J.-C. Leroux, *Sci. Adv.*, 2021, **7**, abe9499.
